# Incidence of Barotrauma in COVID-19 Patients Requiring Mechanical Ventilation: A Retrospective Study in a Community Hospital

**DOI:** 10.7759/cureus.30233

**Published:** 2022-10-12

**Authors:** Onkar Mudhar, Sanjeev K Goswami, Jacquie DeMellow

**Affiliations:** 1 Internal Medicine, St. Joseph’s Medical Center, Stockton, USA; 2 Pulmonary and Critical Care Medicine, St. Joseph’s Medical Center, Stockton, USA; 3 Quality Improvement/Critical Care, St. Joseph’s Medical Center, Stockton, USA

**Keywords:** protective ventilation, ards (acute respiratory distress syndrome), covid-associated pneumothorax, pulmonary barotrauma, covid-19

## Abstract

Background and aim

Acute respiratory distress syndrome (ARDS) is a severe complication of COVID-19 and traditional ventilation strategies using ARDSNet protocol, including low tidal volumes, appear to cause barotrauma in COVID-19 patients at a higher rate than non-COVID-19 ARDS patients. The purpose of our study was to determine if COVID-19 patients with ARDS undergoing mechanical ventilation at St. Joseph's Medical Center (SJMC) developed barotrauma at a higher rate than non-COVID-19 ARDS patients.

Methods and materials

This study was a retrospective chart review of all patients admitted to critical care units at SJMC with COVID-19 infection and requiring mechanical ventilation from March 1, 2020 to September 30, 2020. The sample included adult patients (aged 18 and above) with the International Classification of Diseases (ICD) 10 code for COVID-19 (U07.1) and patients who were placed on mechanical ventilation for longer than 24 hours, from March 1, 2020 to September 30, 2020. Barotrauma was confirmed via radiographic imaging including chest X-ray, CT, or CT angiography (CTA).

Results

One hundred and forty COVID-19 patients underwent mechanical ventilation for longer than 24 hours from March 1, 2020 to September 30, 2020 at our facility. Twenty-six COVID-19 patients (18.6%) met our inclusion criteria (development of barotrauma during hospital admission) of which 25 patients (17.9%) underwent mechanical (invasive and/or non-invasive) ventilation prior to the development of barotrauma. Around 80% of the patients were on non-invasive mechanical ventilation prior to intubation and invasive mechanical ventilation. The categorical breakdown of barotrauma was as follows: pneumothorax 65.4%, subcutaneous emphysema 61.5%, pneumomediastinum 34.6%, and pneumoperitoneum 7.7%. None of the included patients had any previous history of documented barotrauma. Prior to the time of barotrauma, 17 patients were on volume control, seven were on pressure control, and one was not on mechanical ventilation. Of the 17 patients on volume control, only one patient was above the ARDSNet guideline of 6-8 mL/kg ideal body weight (IBW). In comparison to ARDS patients at SJMC in 2019, only two out of 28 patients (7.14%) developed barotrauma during mechanical ventilation.

Conclusions

Patients with COVID-19 who underwent mechanical ventilation developed barotrauma at a higher rate than traditional non-COVID-19 patients with ARDS.

## Introduction

The first cases of COVID-19 were discovered in Wuhan, China in December 2019 and the Centers for Disease Control and Prevention (CDC) confirmed the first case in the United States on January 20, 2020 [1]. This disease quickly plunged China into an epidemic with a subsequent worldwide pandemic. What was striking about this disease was the severity of the respiratory symptoms in the patient population. Most notably, patients with COVID-19 undergoing mechanical ventilation appeared to be developing barotrauma at a higher rate than patients with non-COVID-19 acute respiratory distress syndrome (ARDS). A study done at the New York University (NYU) Langone Health found that during their study period, 15% of their COVID patients developed barotrauma vs 0.5% of their non-COVID patients; their overall barotrauma rate in ARDS patients over the four years before the COVID-19 pandemic was 11% [2]. Another study in Italy found similar results with pneumomediastinum or subcutaneous emphysema occurring in 13.6% of their COVID-19 ARDS patients undergoing mechanical ventilation vs 1.9% in non-COVID ARDS patients [3]. The exact etiology of this increased rate of barotrauma is not fully understood and despite using lung-protective ventilation which ARDSnet has defined as 6 mL/kg to 8 mL/kg ideal body weight (IBW), COVID-19 patients continue to develop barotrauma at a rate not seen before in traditional ARDS patients [4]. The purpose of our study was to determine if this higher rate of barotrauma was also present in COVID-19 ARDS patients at St. Joseph's Medical Center (SJMC) and add to the growing database of literature supporting these findings.

## Materials and methods

The SJMC electronic medical record (EMR) was searched for adult patients (aged over 18) admitted with International Classification of Diseases (ICD) 10 code for COVID-19 (U07.1) and patients who were placed on mechanical ventilation for longer than 24 hours, between March 1, 2020 and September 30, 2020. A total of 140 COVID-19 patients were found to have met the above criteria. A chart review was then conducted with a review of progress notes as well as all imaging studies of each patient obtained during the admission period to identify cases involving barotrauma. Barotrauma was classified into five separate categories: pneumothorax, pneumomediastinum, subcutaneous emphysema, pneumopericardium, and pneumoperitoneum.

Of the 140 COVID-19 patients undergoing mechanical ventilation during our study period, 26 patients (18.6%) met our inclusion criteria (development of barotrauma during hospitalization) of which 25 (17.9%) underwent mechanical (invasive and/or non-invasive) ventilation prior to developing barotrauma. Non-Invasive ventilation was defined as bilevel positive airway pressure (BiPAP)/continuous positive airway pressure (CPAP) whereas invasive ventilation was defined as endotracheal intubation with subsequent ventilation. Utilization of high flow nasal cannula (HFNC) prior to invasive mechanical ventilation was also noted. None of the 26 patients who met our inclusion criteria had a previous history of barotrauma.

Additional demographic information was collected including age, sex, race, comorbidities, height, weight, and body mass index (BMI). Ideal body weight (IBW) was calculated utilizing the formula 50 kg + 2.3 kg x (height in inches - 60) for males and 45.5 kg + 2.3 kg x (height in inches - 60) for females. Ventilation-specific data including start date/time, arterial partial pressure of oxygen/fraction of inspired oxygen (P/F) ratio on the day of barotrauma, positive end-expiratory pressure (PEEP)/fraction of inspired oxygen (FiO2) at the time of barotrauma, and ventilation mode at the time of barotrauma was also collected. Plateau pressures at the time of barotrauma were unable to be obtained for all 26 patients due to limitations in the documentation.

The COVID-19 ARDS barotrauma rates were compared to SJMC’s 2019 ARDS barotrauma rate. An ICD 10 code search for ARDS and barotrauma was utilized in determining SJMC’s 2019 barotrauma rate for ARDS patients undergoing mechanical ventilation.

This retrospective study was approved (approval no. CANV DHIRB-2020-665) by the Institutional Review Board (IRB) of SJMC.

## Results

Twenty-six patients developed barotrauma during our study period and barotrauma was defined as pneumothorax, subcutaneous emphysema, pneumomediastinum, and pneumoperitoneum. Baseline characteristics are shown in Table 1. 

**Table 1:** Baseline patient characteristics at the time of barotrauma BMI: Body mass index, COPD: Chronic obstructive pulmonary disease, OSA: Obstructive sleep apnea, CHF: Congestive heart failure

Variables	Average with percentage or range
Age	61 (23 – 82)
Male	16 (62%)
Female	10 (38%)
Caucasian	12 (46%)
African American	2 (8%)
Asian	1 (4%)
Other	11 (42%)
Weight (kg)	86.35 (63.8 – 146.9)
BMI	31.3 (18.8 – 52.3)
COPD	3 (12%)
OSA	6 (23%)
CHF	5 (19%)
Atrial fibrillation	2 (8%)

The categorical breakdown of barotrauma was as follows: pneumothorax 65.4%, subcutaneous emphysema 61.5%, pneumomediastinum 34.6%, and pneumoperitoneum 7.7%. Our overall barotrauma rate during the study period was found to be 18.6%. A review of the EMR did not reveal a previous history of barotrauma in all 26 patients which met our inclusion criteria.

Table 2 summarizes the patient care as well as ventilation settings at the time of barotrauma. Notably, one patient was never on mechanical ventilation but was incidentally found to have developed a pneumothorax on a post-thoracentesis chest X-ray. Two patients were found on imaging to have developed barotrauma while on non-invasive mechanical ventilation before initiating invasive mechanical ventilation. One of these patients was on high flow nasal cannula (HFNC) for eight days with subsequent BiPAP for five days prior to intubation, and the other patient was on HFNC for seven days and subsequent BiPAP for one day before intubation. The average time on HFNC or BiPAP/CPAP prior to invasive mechanical ventilation was 2.72 days and 3.9 days, respectively. The timing of central line placement in relation to barotrauma development was also noted due to the inherent risk of barotrauma associated with the line placement, and in our study, five patients were found to have had a central line placed (internal jugular (IJ), subclavian, or peripherally inserted central catheter (PICC)) within 24 hours prior to barotrauma development. It is worth noting that four out of five of these patients also had invasive mechanical ventilation initiated on the day of barotrauma development.

**Table 2:** Patient care variables at the time of barotrauma * Three patients out of 26 had a Δ PEEP in the 24 hours prior to developing barotrauma. Two patients had an increase of 5 and one patient had an increase of 6 ** Plateau pressure documented in 14 of 26 patients at the time of barotrauma *** Average calculated using set tidal volume documented in 17 of 26 patients who were on a volume control ventilation mode at the time of barotrauma **** Percentages calculated utilizing the total of 23 patients who were on invasive mechanical ventilation prior to the development of barotrauma RASS: Richmond agitation sedation scale, HFNC: High flow nasal cannula, BiPAP: Bilevel positive airway pressure, CPAP: Continuous positive airway pressure, P/F ratio: Arterial partial pressure of oxygen (PaO2)/fraction of inspired oxygen (FiO2), PEEP: Positive end-expiratory pressure, PIP: Peak inspiratory pressure, IBW: Ideal body weight

Variables	Value with percentage or range if applicable
On a neuromuscular blocker	15.40%
Currently/previously on corticosteroids	96.20%
Undergoing proning	42.30%
On sedation drip (RASS -3 to -5)	92.30%
On noninvasive ventilation prior to intubation	81%
Average number of days on HFNC prior to intubation	2.72 (0-8)
Average number of days on BiPAP/CPAP prior to intubation	3.91 (0-23)
Barotrauma prior to intubation	1 (4%)
Barotrauma day of intubation	10 (38%)
Barotrauma post intubation	14 (54%)
Average P/F ratio at the time of barotrauma (PaO2/FiO2)	94 (41 - 252)
24 hour change in PEEP (cm of H20)	0*
Average PEEP (cm of H20)	13 (8 - 20)
Average PIP (cm of H20)	34.5 (24-46)
Average plateau pressure (cm of H20)	32.5 (25 - 46)**
Average tidal volume (cc/kg of IBW)	409 (300 - 500)***
Volume control ventilation mode	17 (74%)****
Pressure control ventilation mode	6 (26%)****

**Figure 1:**
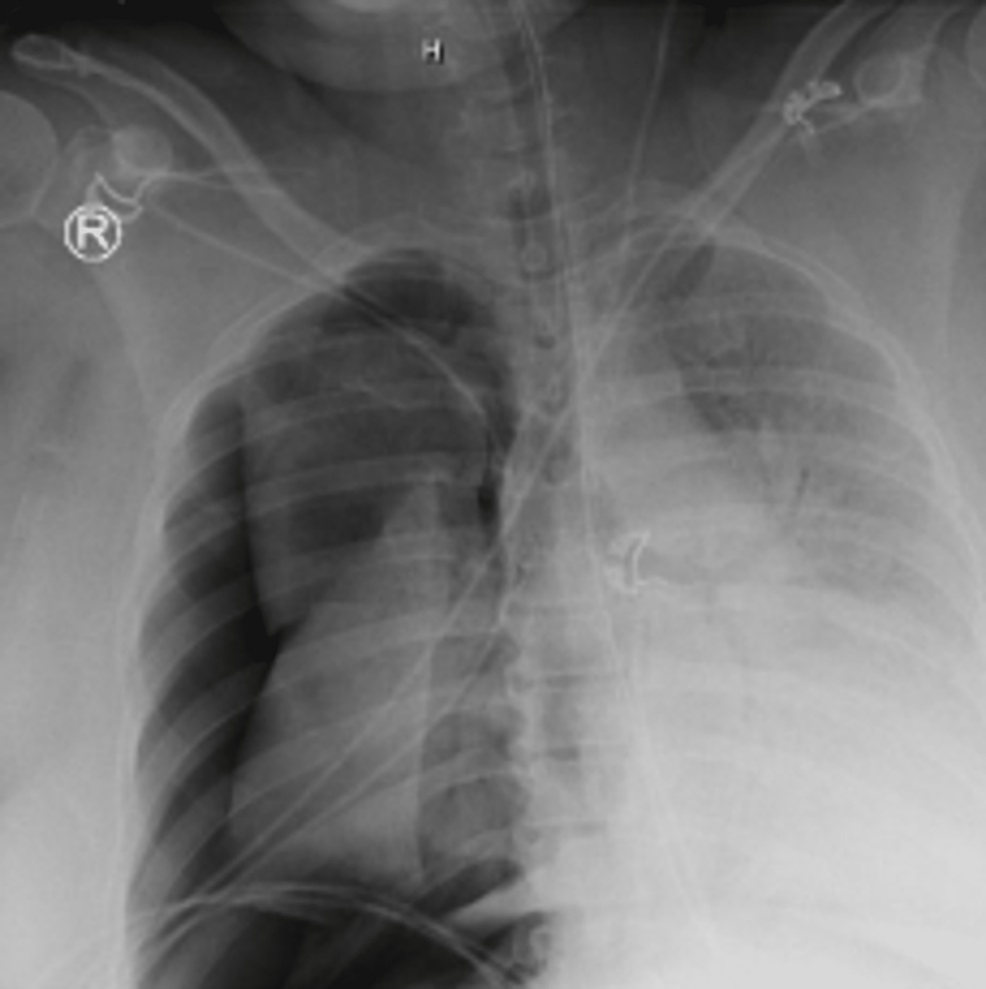
Imaging of a female patient in her 50s who developed a 50% right-sided pneumothorax on the day she was initiated on mechanical ventilation.

**Figure 2:**
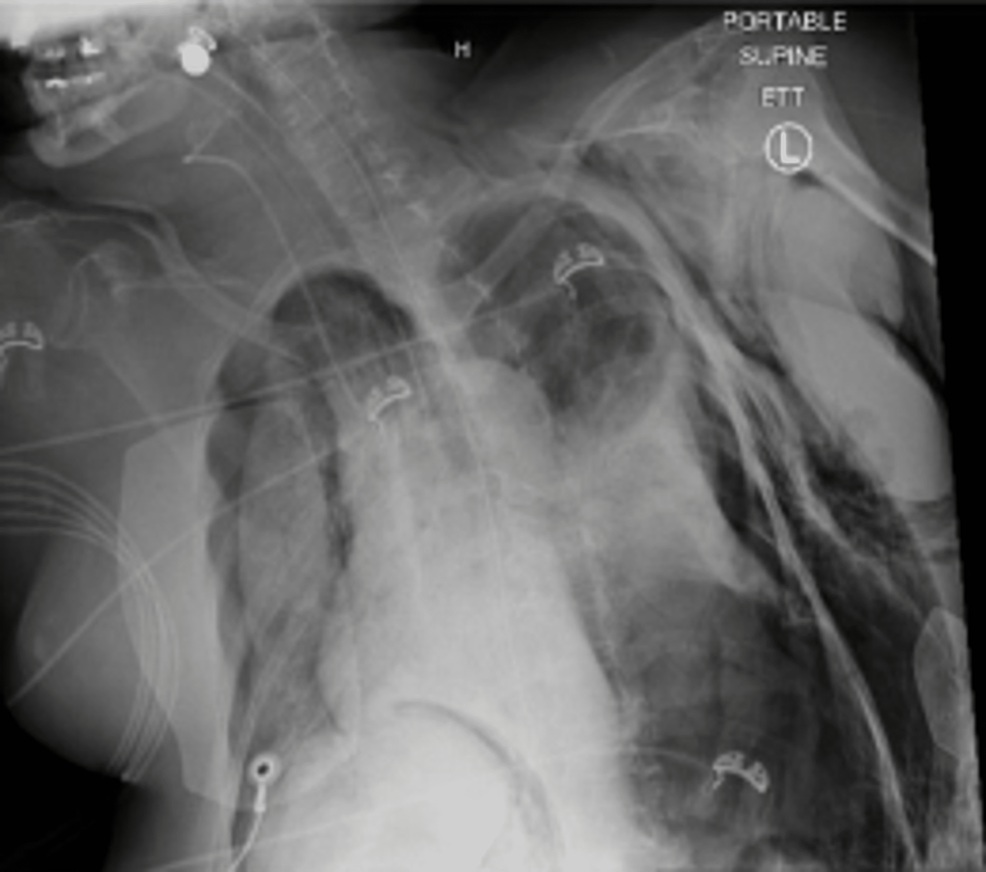
Imaging of a female patient in her 60s who developed bilateral pneumothoraces, right greater than left, along with left chest wall subcutaneous emphysema and pneumoperitoneum.

The vast majority of patients developed barotrauma either on the day of intubation or in the days immediately following intubation. Notably, 81% of patients were on noninvasive mechanical ventilation (CPAP/BiPAP) prior to undergoing intubation and mechanical ventilation. Two patients in our study were briefly trialed on noninvasive mechanical ventilation prior to being intubated and initiated on invasive mechanical ventilation on the day of admission. The IBW was calculated for all 26 patients included in the study and out of 17 patients who were on a volume control mode, only one was above the ARDSNet guideline of 6 mL/kg to 8 mL/kg IBW at the time of barotrauma. Six patients were on a pressure control mode at the time of barotrauma and due to the inherent variability of ventilation volumes associated with a pressure control mode, returned tidal volumes at the time of barotrauma were documented; two out of the six patients on a pressure control mode were noted to have a returned tidal volume greater than the ARDS upper limit of 8 mL/kg IBW, while the remaining four patients were underneath this limit. Plateau pressures were unable to be captured for all 26 patients that met our inclusion criteria due to limitations in documentation, however, peak inspiratory pressures (PIP) were documented for our entire sample population; four patients were found to have a PIP of 40 cm/H20 or greater. Of the 14 patients where plateau pressure was successfully captured, seven patients had a plateau pressure >30 at the time of barotrauma and seven patients had a plateau pressure <30. The extent of barotrauma development in our patient population is illustrated in Figures 1-2. 

## Discussion

Barotrauma is a known complication of mechanical ventilation and the incidence of barotrauma in ARDS patients undergoing mechanical ventilation varies anywhere from 4.8% to 11% [5]. However, with COVID-19 the rates appear to be higher and this is confirmed via multiple studies across the world. A study conducted at NYU Langone Health in 2020 examined the barotrauma rate with COVID-19 patients undergoing invasive mechanical ventilation. Six hundred and one patients with COVID-19 underwent invasive mechanical ventilation during the study period and 89 patients (15%) experienced one or more barotrauma events [2]. During the same period at NYU Langone Health, 196 patients without COVID-19 on invasive mechanical ventilation had one barotrauma event (0.5%) [2]. The overall ARDS barotrauma rate for NYU Langone Health four years prior to this study was 11%, indicating a statistically significant increase in the barotrauma rate in COVID-19 patients undergoing invasive mechanical ventilation [2]. Another study conducted at the Fondazione Poliambulanza Hospital between February 18 and April 15, 2020, found similar results; during the study period, pneumomediastinum/subcutaneous emphysema occurred in 23 out of 169 (13.6%) patients with COVID-19 ARDS and three out of 163 patients (1.9%) with non-COVID-19 ARDS [3]. A meta-analysis conducted in 2022 identified 4488 studies of COVID-19 barotrauma, with barotrauma occurring in 4.2% of hospitalized patients, 15.6% of critically ill patients, and 18.4% of patients undergoing invasive mechanical ventilation, indicating a linear relationship of barotrauma with the severity of the disease [6]. Literature has consistently shown that barotrauma occurs at a higher rate in COVID-19 mechanically ventilated patients than in traditional ARDS ventilated patients and the results of our study are consistent with these literature findings. 

During our study period, SJMC had a barotrauma rate of almost 18% in ventilated COVID-19 ARDS patients. Compared with ARDS patients undergoing mechanical ventilation at SJMC in 2019, only two out of 28 patients (7.14%) developed barotrauma. The exact cause of the increased rate of barotrauma in COVID-19 ARDS patients is currently unknown, however, several theories are beginning to emerge. A study conducted by Fox et al. in New Orleans examined the autopsies of 10 African American patients with the cause of death attributed to COVID-19. Histological examination of lung tissue revealed diffuse alveolar damage along with thrombosed small vessels and associated mononuclear response and hemorrhage [7]. This pattern of alveolar damage is similar to what was discovered in autopsy reports from the first severe acute respiratory syndrome (SARS) epidemic but the severe burden of these findings is a step above what has previously been seen and may help explain why COVID-19 barotrauma has been so severe [7]. The extent of the damage may make the alveolar wall more prone to rupture and it can be exacerbated by cough or anything which increases the alveolar pressure including noninvasive ventilation [8]. We hypothesize that noninvasive ventilation may contribute to the weakening of the pulmonary architecture and when patients are eventually intubated and switched to invasive mechanical ventilation, alveolar pressures increase to the point of causing barotrauma, even in traditional lung protective ventilation settings. In our study, out of 17 patients who were on a volume control mode only one patient had a tidal volume above the ARDSNet guideline of 8 ml/kg IBW, and for the six patients on a pressure control mode, two patients had a returned tidal volume above the ARDSNet guideline. It appears that despite overall appropriate ventilation volumes, high sedation levels as well as the use of paralytics, there were multiple instances of PIP >40 and plateau pressures >30, suggesting an overarching difficulty in ventilation of COVID-19 ARDS patients. Our small sample population has demonstrated instances of barotrauma occurring even before invasive mechanical ventilation is initiated and maintaining patients on noninvasive ventilation for prolonged periods before intubation may have contributed to the diffuse alveolar damage associated with COVID-19 and subsequently more instances of barotrauma. As there have been multiple reports of patients developing barotrauma even off of mechanical ventilation, care must be taken when considering mechanical ventilation for COVID-19 patients as it appears the extent of pulmonary damage this disease is causing is more severe than what has been previously seen in traditional ARDS. 

There are several limitations to this study, most notably its small sample size and that it is a single-center retrospective study. We were also limited in the data that was collected at the time of barotrauma, specifically in regards to plateau pressure, which may have been in part due to the urgent pathology that was rapidly progressing with our patient population as well as the strains placed upon our hospital during the COVID-19 surges. We have attempted to mitigate the limitations created by incomplete plateau pressure data by capturing data on PIP and tidal volumes for our patient population.

The strengths of this study are its generalizability as our findings are consistent with studies across the world that examined barotrauma in COVID-19 ventilated patients. As hospitals are tracking adverse events that occurred during the pandemic, statistics regarding barotrauma can likely be obtained in the vast majority of hospitals throughout the globe. 

Existing literature confirms many of the findings that have been presented in our study but we hope that our data will help bring light to ICU-level care that was administered to COVID-19 patients in a community-based setting during the pandemic as well as further discussion regarding the optimization of the duration of noninvasive ventilation, ventilation volumes, and inspiratory pressures in COVID-19 ARDS patients.

## Conclusions

The results of this study suggest that patients with COVID-19 who underwent lung protective mechanical ventilation at SJMC developed barotrauma at a higher rate than non-COVID-19 patients with ARDS. These findings are consistent with literature that has been published throughout the COVID-19 pandemic, however, the exact mechanism behind this higher rate of barotrauma is currently unknown. Further research is required to determine optimal ventilation volumes, pressures, and durations in COVID-19 ARDS patients as these patients do not appear to fall in line with current ARDS ventilation guidelines. 
